# A four-coordinate cobalt(II) single-ion magnet with coercivity and a very high energy barrier

**DOI:** 10.1038/ncomms10467

**Published:** 2016-02-17

**Authors:** Yvonne Rechkemmer, Frauke D. Breitgoff, Margarethe van der Meer, Mihail Atanasov, Michael Hakl, Milan Orlita, Petr Neugebauer, Frank Neese, Biprajit Sarkar, Joris van Slageren

**Affiliations:** 1 Institut für Physikalische Chemie, Universität Stuttgart, Pfaffenwaldring 55, Stuttgart D-70569, Germany; 2 Institut für Chemie und Biochemie, Anorganische Chemie, Freie Universität Berlin, Fabeckstraße 34-36, Berlin D-14195, Germany; 3 Max Planck Institute for Chemical Energy Conversion, Stiftstraße 34-36, Mülheim an der Ruhr D-45470, Germany; 4 Institute of General and Inorganic Chemistry, Bulgarian Academy of Sciences, Sofia 1113, Bulgaria; 5 Laboratoire national des champs magnétiques intenses, CNRS-UJF-UPS-INS, Grenoble F-38042, France; 6 Institute of Physics, Charles University in Prague, Prague CZ-12116, Czech Republic

## Abstract

Single-molecule magnets display magnetic bistability of molecular origin, which may one day be exploited in magnetic data storage devices. Recently it was realised that increasing the magnetic moment of polynuclear molecules does not automatically lead to a substantial increase in magnetic bistability. Attention has thus increasingly focussed on ions with large magnetic anisotropies, especially lanthanides. In spite of large effective energy barriers towards relaxation of the magnetic moment, this has so far not led to a big increase in magnetic bistability. Here we present a comprehensive study of a mononuclear, tetrahedrally coordinated cobalt(II) single-molecule magnet, which has a very high effective energy barrier and displays pronounced magnetic bistability. The combined experimental-theoretical approach enables an in-depth understanding of the origin of these favourable properties, which are shown to arise from a strong ligand field in combination with axial distortion. Our findings allow formulation of clear design principles for improved materials.

## Abstract (web summary)

Bistable single-molecule magnets potentially allow information storage at extremely high densities. Here, the authors study an air- and moisture-stable mononuclear tetrahedral cobalt(II) complex, elucidating the origin of its pronounced magnetic bistability.

## Graphical abstract

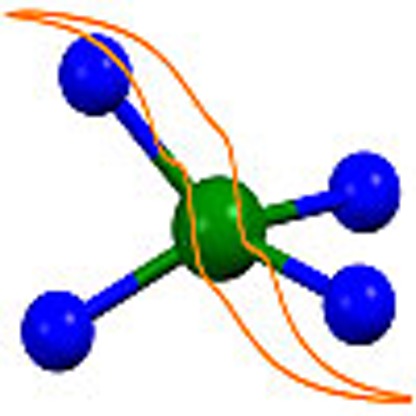

Single-molecule magnets (SMMs) are molecules that display slow relaxation of the magnetization of purely molecular origin^1^,^2^. A molecule with a bistable magnetization may be used to store information, with one orientation of the magnetic moment encoding a binary 0, the opposite orientation a 1. The origin of the bistability is the presence of an energy barrier between the up and down orientations of the magnetic moment. Traditionally, mostly polynuclear coordination complexes of first row transition metals have been investigated in this respect. For such systems, the effective energy barrier *U*_eff_ is given by *U*_eff_=*DS*^2^ for molecules with integer spin ground states and *U*_eff_=*D*(*S*^2^−¼) in the half-integer case. Here *S* is the spin of the ground state of the molecule and *D* is the magnetic anisotropy constant, more precisely, the second rank axial zero-field splitting (ZFS). For many years, research efforts focussed on increasing the spin of the ground state and spin values of over 40 were achieved^3^. In spite of these efforts, it took 14 years to increase the energy barrier from 43 cm^−1^ (=62 K) for [Mn_12_O_12_(OAc)_16_(H_2_O)_4_] to 60 cm^−1^ (=86 K) for [Mn_6_O_2_(sao)_6_(O_2_CPh)_2_(EtOH)_4_] (refs 4, 5). The reason for this slow progress is that with increasing *S*, *D* actually goes down and the energy barrier does not improve much^6^,^7^,^8^. Hence, attention turned to the other parameter in the equation, namely the magnetic anisotropy. This has led to an explosion in interest in ions with large magnetic anisotropies. Arguably the strongest interest has been in complexes of the lanthanides, where energy barriers of almost 1,000 K have been reported^9^,^10^. However, in these complexes, there is typically no bistability of the magnetization that would be evidenced by substantial coercivity in the magnetic hysteresis curve. To prevent this, one has to develop strongly magnetically coupled polynuclear complexes^11^. This has so far only been achieved in highly air-sensitive complexes with radical ligands^12^,^13^. Without radical bridges, strong couplings are very difficult to obtain in lanthanide-based systems. In transition metal chemistry, magnetic couplings are much easier to achieve and these ions are enjoying renewed interest as a result. In this context, attention focusses on high-anisotropy transition metal ions and it has been discovered that in a number of cases slow relaxation of the magnetization can be observed for mononuclear complexes, also called single-ion magnets^14^,^15^,^16^,^17^. However, so far the effective energy barriers have been typically modest (of the order of 30 cm^−1^), and in most cases it proved to be necessary to apply a small direct current magnetic field to suppress tunnelling and observe slow relaxation of the magnetization (field-induced single-ion magnet). Currently, there is only one example of a zero-field SMM with an energy barrier that exceeds 200 cm^−1^, and this is the linear iron(I) complex [K(crypt-222)][Fe(C(SiMe_3_)_3_)_2_] with an energy barrier of *U*_eff_=226 cm^−1^ (ref. 18). However, this complex is rather air-sensitive, hindering possible practical application. A further difficulty is the possible presence of a first order orbital angular momentum, which complicates the analysis. In contrast, the d^7^ ground term of cobalt(II) in *T*_d_ symmetry is ^4^A_2_. Hence tetrahedral cobalt(II) is a pure spin ion, and the spin-Hamiltonian model is appropriate for the analysis. Rather large values of the ZFS parameter *D* (described by the spin-Hamiltonian H=*DŜ*_z_^2^) have been reported for tetrahedral cobalt(II), with six examples of |*D*|>50 cm^−1^ (refs 19, 20, 21, 22, 23, 24).

Here we present results of our investigations of the mononuclear tetrahedral cobalt(II) complex (HNEt_3_)_2_[Co^II^(L^2−^)_2_] (**1**), where H_2_L=1,2-bis(methanesulfonamido)benzene. We show that this synthetically flexible, fully air- and moisture-stable complex has both a ZFS and an energy barrier exceeding 200 cm^−1^. It shows magnetic hysteresis with a coercive field up to 0.24 T at 1.5 K and a sweep rate of 0.5 T min^−1^. By the combined results of spectroscopic and theoretical methods, the unique magnetic properties are shown to arise from the strong axial ligand field produced by the bis(sulfonamide) ligands, leading to a small energy gap between the ^4^B_1_ ground state and the ^4^B_2_ first excited state of Co(II). Our findings not only completely unravel the origin of the advantageous properties of complex **1**, but also allow for the derivation of guidelines for further improvement.

## Results

### Synthesis and structure

**Figure 1:**
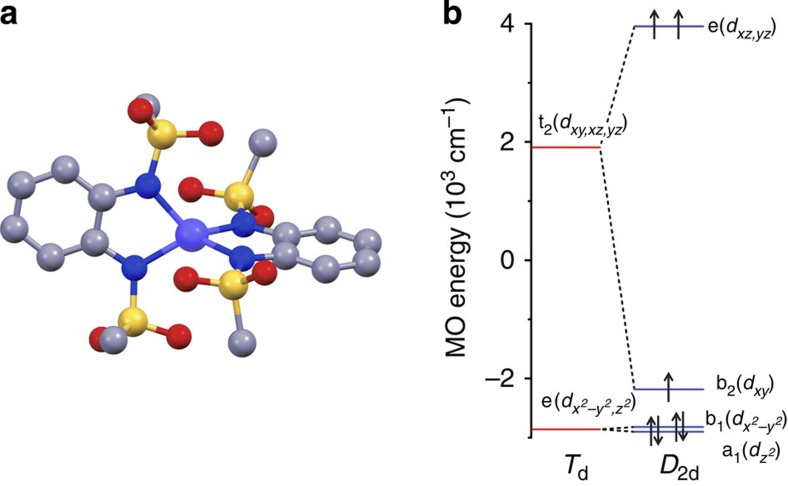
Molecular and electronic structure of complex 1. (**a**) Crystallographic structure of the anion of **1**. Cobalt is shown in blue, oxygen in red, sulfur in yellow, nitrogen in violet and carbon in grey. Hydrogen atoms have been omitted for clarity. (**b**) Molecular orbital diagram showing the calculated d-orbital splitting for **1**. Horizontal lines depict orbital energies while arrows pointing up or down stand for single electron spins.

Complex **1** was synthesized at room temperature by the reaction of Co(BF_4_)_2_·6H_2_O and H_2_L in acetronitrile in the presence of NEt_3_ as a base. Diffusion of diethylether into the reaction mixture delivered the pure, crystalline complex in about 80% yield. The crystallographic structure (Fig. 1a and Supplementary Data set 1) shows the cobalt ion bound to two doubly deprotonated 1,2-bis(methanesulfonamido)benzene ligands oriented perpendicularly to each other, resulting in a distorted tetrahedral coordination geometry for the cobalt ion. The C–N distances within the ligand in **1** are in the range 1.409–1.418 Å and the intra-ring C–C distances within the ligand are in the range 1.378–1.421 Å. These distances clearly point to the existence of C–N single bonds and an aromatic ring^25^,^26^, thus supporting the best formulation of this complex as [Co^II^(L^2−^)_2_]^2−^. The overall 2– charge for the complex is compensated by two NHEt_3_ cations (Supplementary Fig. 1). Compound **1** crystallizes in the orthogonal P2_1_2_1_2_1_ space group with four molecules in the unit cell, all symmetry-related by the four mutually orthogonal twofold rotoinversion axes. As a result the site symmetry of the complex is *C*_1_. However, the idealized point group symmetry is *D*_2d_. Thus, the least squares planes defined by the Co-NCCN metallacycles are almost perpendicular with an angle of 84.83°. The two N–Co–N angles for the two ligands are 80.59° and 80.70°, that is, virtually the same but much smaller than the ideal 109.5° for a perfect tetrahedron. All Co–N bond lengths are very similar and range from 1.998 to 2.013 Å (Supplementary Fig. 2, Supplementary Table 1).

### Magnetic properties and far-infrared spectroscopy

**Figure 2:**
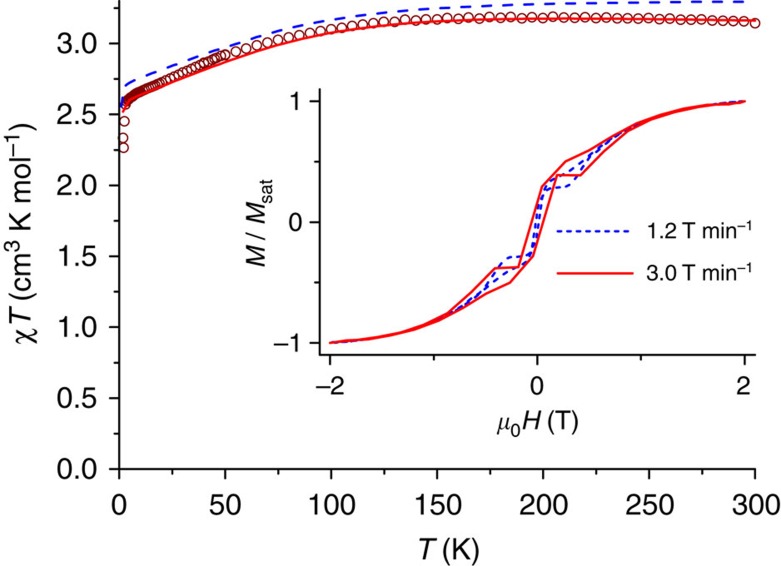
DC susceptibility. Susceptibility temperature product *χT* as a function of temperature recorded on a powder sample of **1** at an applied field of 1,000 Oe (*T*<50K) and 10,000 Oe (*T*>40 K). The solid red line is a spin-Hamiltonian fit with *D*=–115 cm^−1^, *g*_⊥_=2.20, *g*_||_=3.03. The dashed blue line is a simulation on the basis of the correlated calculations. The inset shows the magnetic hysteresis curve at *T*=1.8 K for **1** in fluorolube at two different scan rates as indicated.

Figure 2 shows the static magnetic susceptibility temperature product *χT* as a function of temperature and the magnetization as a function of field. The room temperature *χT* value of 3.14 cm^3^ K mol^−1^ corresponds to the value expected for an *S*=3/2 ion with *g*=2.59. Similar values have been reported for other cobalt(II) complexes^15^,^19^,^20^,^27^. On decreasing the temperature, the *χT* value remains essentially constant down to 130 K, below which it decreases. We attribute this decrease to the presence of a large ZFS. The decrease is much too pronounced to be attributable to intermolecular interactions, because the Co–Co distance is far too large (8.651 Å). The magnetization of the sample at 7 T and 1.8 K is *M*=2.56 *μ*_B_ (Supplementary Figure 3), which is far below the expected saturation value for *S*=3/2 and *g*=2.59 (*M*_sat_=3.9 *μ*_B_) and is another indication of large ZFS. Indeed, both the susceptibility and the magnetization data can be fitted well with *D*=–115±20 cm^−1^ and *g*_⊥_=2.20±0.05, *g*_||_=3.03±0.03. Fits with isotropic *g* values did not lead to a satisfactory result. This *D* value is the second largest reported for four-coordinate cobalt(II) (Table 1). As a result of the negative sign of *D*, the *m*_S_=±3/2 Kramers doublet (KD) is lowest in energy. It is separated from the *m*_S_=±1/2 doublet by an energy gap of 2*D*, in this case therefore *ca.* 230 cm^−1^. Because at 1.8 K the excited KD is not populated, the magnetization curve can also be fit by means of a pseudo spin *S*=½ and *g*_⊥_=0, *g*_||_=9.1±0.1 (Supplementary Fig. 3).

**Figure 3:**
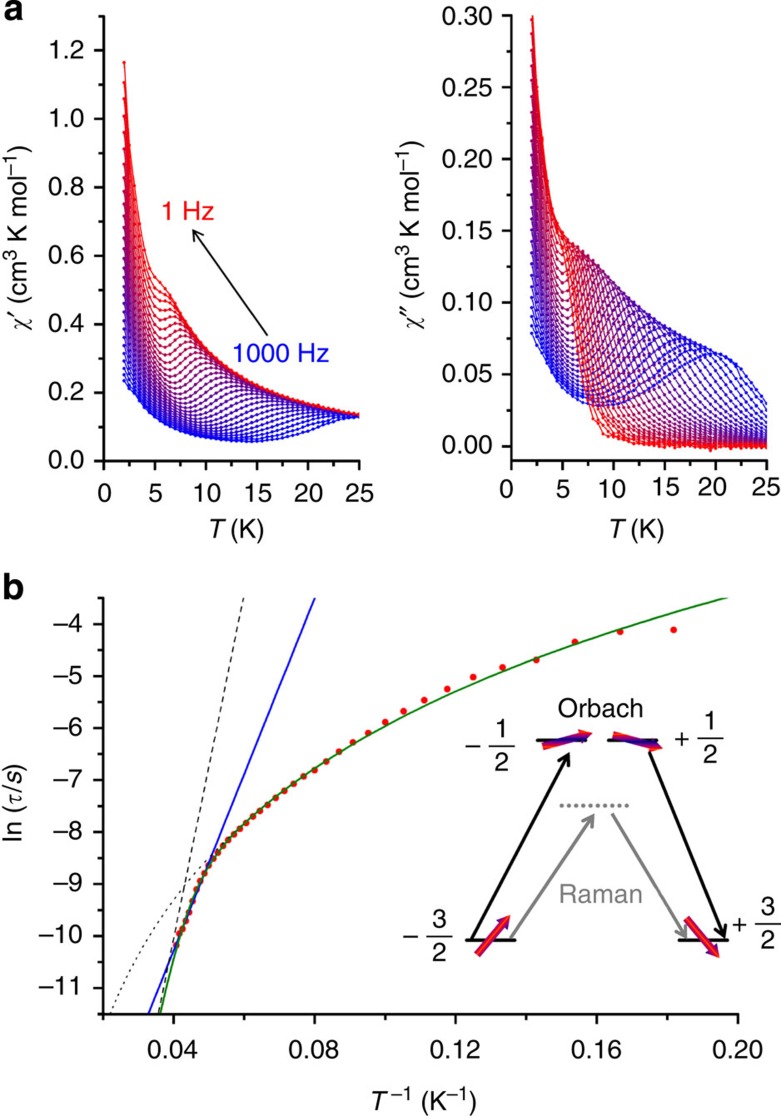
AC susceptibility data and Arrhenius plot for 1. (**a**) In-phase *χ*′ and out-of-phase components *χ*″ of the ac susceptibility as a function of temperature recorded on a pressed powder sample of **1** in zero dc field and at different frequencies of the ac magnetic field as indicated. (**b**) Natural logarithm of the relaxation time ln *τ* as a function of the inverse temperature *T*^−1^. The straight blue line is a fit of the 10 points at highest temperatures to the Arrhenius law ln *τ*=ln *τ*_0_+*U*_eff_/*k*_B_*T* with *τ*_0_=3.89 × 10^–8^ s and *U*_eff_=118 cm^−1^. The curved green line is the fit to the sum of Raman and Orbach processes *τ*^−1^=*CT*^*n*^+*τ*_0_^−1^ exp(–*U*_eff_/*k*_B_*T*) with *C*=0.087, *n*=3.65, *τ*_0_^−1^=1.31 × 10^10^ s^−1^ and *U*_eff_=230 cm^−1^ (fixed). The dashed and dotted grey lines are the Orbach and Raman contributions, respectively. The insert shows the energies of the *m*_S_ states as a function of the *m*_S_ quantum number and schematically displays the Orbach and Raman relaxation pathways. Horizontal lines correspond to energy levels and red arrows illustrate the magnetic moment.

Mononuclear four-coordinate cobalt(II) complexes have been reported to show slow relaxation of the magnetization^14^,^21^,^24^,^28^,^29^. To investigate the magnetization dynamics, we carried out magnetic hysteresis measurements (Fig. 2). At the lowest temperature accessible to us (1.8 K), and a moderate sweep rate of 200 Oe s^−1^ (1.2 T min^−1^), a typical butterfly-shaped hysteresis curve is obtained, without significant coercivity. Increasing the sweep rate to 500 Oe s^−1^ (3.0 T min^−1^) resulted in opening of the hysteresis curve, with a coercive field of 0.055 T. Furthermore, we performed alternating current (ac) susceptibility measurements at different frequencies and temperatures (Fig. 3, Supplementary Fig. 4). The data show that even without the application of a static external field, complex **1** behaves as a single-molecule magnet. Argand plots of *χ*″ versus *χ*′ (Supplementary Figure 5) were fitted by generalized Debye functions,^2^ to give relaxation rates of the magnetization *τ* at different temperatures. The high *T* data in the Arrhenius plot of ln *τ* as a function of *T*^−1^ (Fig. 3) can be approximated by a straight line in the high-temperature region (10 points), which suggests that at high temperatures relaxation occurs via the excited KD. This corresponds to the Orbach process of spin-lattice relaxation^30^,^31^. At lower temperatures, clearly other relaxation processes, such as Raman, direct and quantum tunnelling processes play a role. The fit of the temperature dependence at high temperatures to the Arrhenius law *τ*=*τ*_0_ exp (*U*_eff_/*k*_B_*T*) yields *U*_eff_=118±5 cm^−1^ and *τ*_0_=3.89 × 10^–8^ s. This effective energy barrier is higher than anything previously reported for four-coordinate cobalt(II) complexes in zero direct current field (Table 1). For mononuclear transition metal single-molecule magnets it is only exceeded by the abovementioned linear Fe(I) complex [K(crypt-222)][Fe(C(SiMe_3_)_3_)_2_] with an energy barrier of *U*_eff_=226 cm^−1^ in zero field^18^. Further large zero-field effective energy barriers reported in the literature include *U*_eff_=71 cm^−1^ (ref. 29), and *U*_eff_=76–103 cm^−1^ (n-Bu_4_N)^+^[Co^II^Co^III^_3_(L)_6_]^−^ (refs 32, 33). Note that in the former case, a higher energy barrier of 152 cm^−1^ was found in a more elaborate analysis (see below). Much higher effective energy barriers have been reported for mononuclear lanthanide complexes^9^, but remanence and coercivity are only observed sporadically in such systems^34^,^35^.

**Figure 4:**
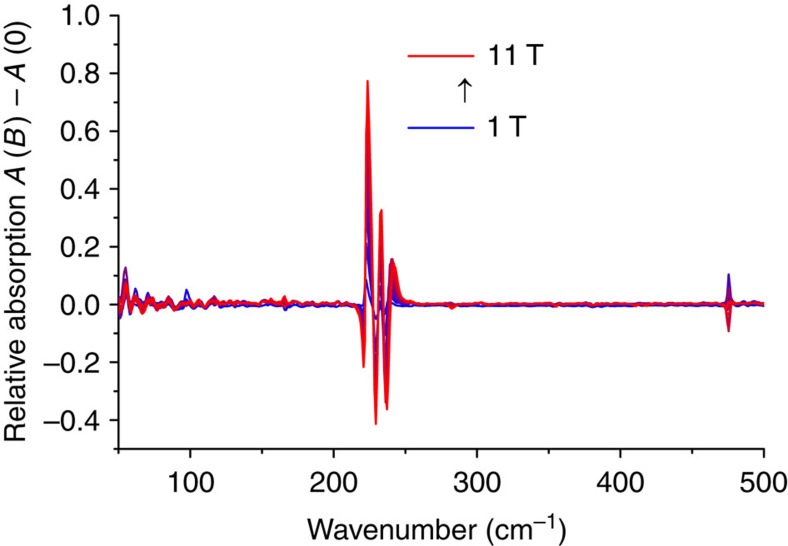
Far-infrared spectroscopy. Normalized far-infrared absorption spectra recorded on a pressed powder sample of **1** at *T*=4 K, obtained by subtracting the zero field absorption spectrum from absorption spectra at different fields.

However, *U*_eff_ is much smaller than the zero-field energy gap, of magnitude 2*D*, predicted by the static magnetic measurements. This means that the derived energy barrier cannot be correct. Similar observations have recently been reported for lanthanides^36^. To resolve this discrepancy, we recorded far-infrared transmission spectra in different magnetic fields (Supplementary Fig. 6), to determine the zero-field gap experimentally. These spectra show a clear field-dependence in the region around 230 cm^−1^. To bring this out more clearly, we have converted the spectra to relative absorption spectra by subtraction of the zero-field absorption spectrum from the absorption spectra at fields of 1–11 T (Fig. 4). Features pointing down are due to the zero-field absorption, features pointing up represent where the spectral density moves in field. The field-dependent features are attributed to the electron paramagnetic resonance transition from *m*_S_=±3/2 to ±1/2. These measurements unequivocally demonstrate the presence of a very high zero-field gap of *ca.* 230 cm^−1^ in **1**, corresponding to |*D|*=115 cm^−1^ (in the absence of a rhombic ZFS term). The resonance line is clearly structured. Simulations based on the Matlab toolbox Easyspin reveal that neither *g* value anisotropy nor rhombic distortion can give rise to the type of splitting observed (Fig. 4). We attribute the structure to spin-vibrational coupling (see below).

With the zero-field energy gap now firmly fixed, we revisit the analysis of the ac susceptibility data. We have fitted the data in Fig. 3b as a sum of the Orbach and Raman processes, given by equation (1), keeping the effective energy barrier fixed at *U*_eff_=230 cm^−1^:


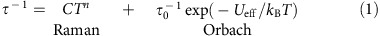


The best fit is obtained for *C*=0.088±0.009, *n*=3.65±0.04, *τ*_0_^−1^=(9.1±0.6) × 10^9^s^−1^. Here we have considered the exponent of the Raman process *n* as a fit parameter. According to the book by Abragam and Bleaney^30^, *n* should be 9 for a Kramers ion (*n*=5 in the presence of low-lying states), but much lower values have been reported (*n*=4–5) (refs 29, 37, 38, 39). One possible reason for this discrepancy is the fact that the derivation that leads to *n*=9 is based on the validity of the Debye model of lattice vibrations, something that has been questioned in literature^40^. Furthermore, it has been shown that the Raman coefficient is lowered when optical rather than acoustic phonons participate in the Raman process^41^. Fit attempts with *n*=9 fixed were not fruitful. This result has the important implication that the energy barrier value derived from a linear fit of the highest temperature relaxation times, even if they clearly lie on a straight line in the Arrhenius plot, may lead to erroneous values. Thus, an energy barrier of *U*_eff_=118 cm^−1^ was derived from a straight-line fit of the 10 highest temperature relaxation points, whereas the true energy barrier is 230 cm^−1^. A similar conclusion was recently reached for a trigonal prismatic cobalt(II) complex, where a straight-line fit to the high-temperature regime yielded *U*_eff_=71 cm^−1^, but a more elaborate fit, taking into account the Raman process, gave *U*_eff_=152 cm^−1^ (ref. 29). It proved to be unnecessary to include contributions due to quantum tunnelling or the direct process. In contrast, for other six-coordinate cobalt(II) complexes, such as [Co(acac)_2_(H_2_O)_2_], no contribution of the Orbach process was found^23^. We attribute the high-energy barrier and relative importance of the Orbach process in **1** to the highly axial nature of the ligand field of **1**. It was reported that highly axial ligand fields reduce the transition matrix elements between the left- and right-hand sides of an energy barrier, thus suppressing the direct and Raman processes^42^. The strongly axial nature of **1** is corroborated by the fact that no EPR lines were observed that could be attributed to transitions within the ground *m*_S_=±3/2 KD, either at conventional or high (300 GHz) frequencies. This transition is forbidden in the absence of rhombic distortion.

### Magnetic circular dichroism spectroscopy

**Figure 5:**
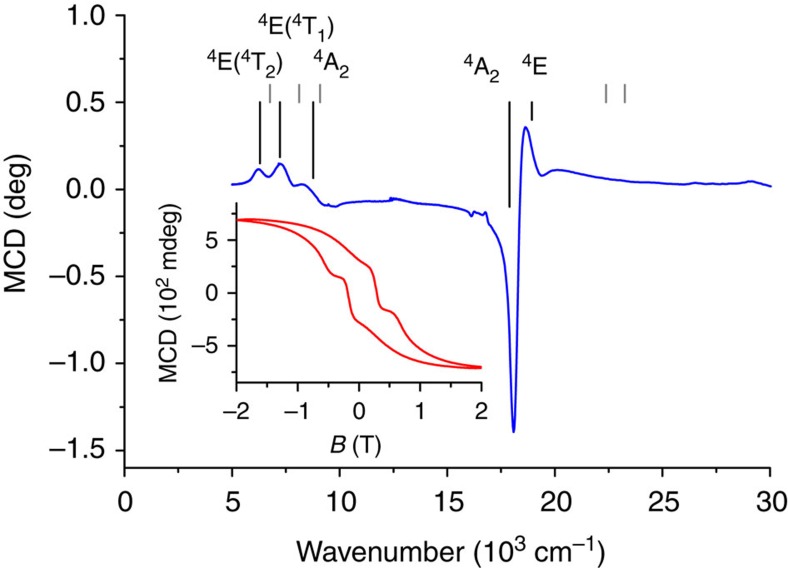
Magnetic circular dichroism spectroscopy. MCD spectra recorded on a mull of **1** in fluorolube at *T*=1.5 K and *B*=2 T. Grey lines are the calculated transition energies from CASSCF calculations, black lines are the transition energies from ligand field analysis of the MCD results. The inset shows the magnetic hysteresis recorded by measuring the MCD intensity at 18,100 cm^−1^ ( that is at maximum of the third ^4^B_1_→^4^E transition band) as a function of the magnetic field *B*, with a scan rate of 0.5 T min^−1^ revealing a coercive field of 0.24 T.

**Figure 6:**
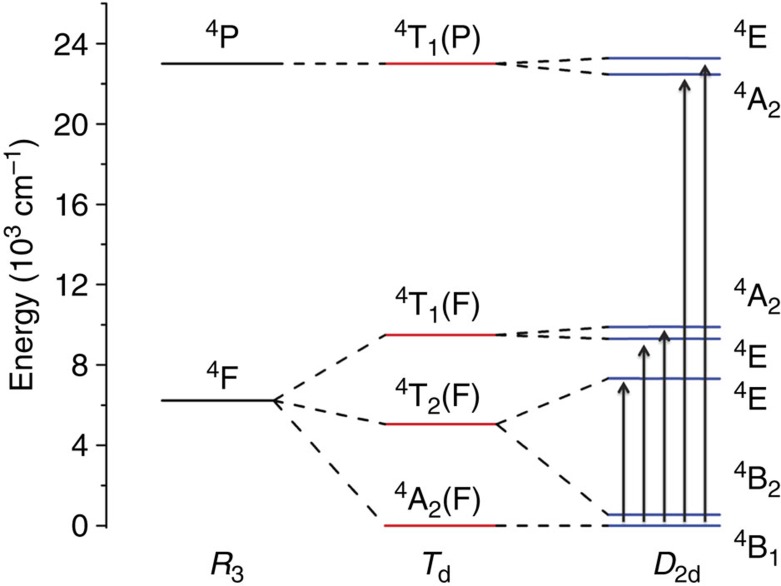
Ligand field splitting. Energy level diagram showing the splitting of the free ion quartet states under the influence of a ligand field with *T*_d_ (red lines) and *D*_2d_ (blue lines) symmetries. The arrows depict the transitions observed in the MCD spectrum.

To link the measured ZFS to the electronic structure of **1**, we recorded electronic absorption and magnetic circular dichroism (MCD) spectra of a mull of **1** at *T*=1.5 K (Fig. 5). In an MCD spectrum, the absorption difference between left- and right-hand circularly polarized light is recorded in the presence of a static magnetic field. Due to the fact that MCD is a signed quantity, resolution is often higher than in ultraviolet/visible spectroscopy. This makes MCD suitable for investigation of (the splitting of) ligand field transitions, which can be connected to the ZFS of an ion.^43^ The ^4^F free ion ground state is split in a ligand field of *T*_d_ symmetry into a ^4^A_2_ ground term with ^4^T_2_(F) and ^4^T_1_(F) excited terms. The next free ion state ^4^P converts into ^4^T_1_(P) in a *T*_d_ symmetry ligand field. In *D*_2d_ symmetry, these terms split according to the energy level diagram of Fig. 6. In the MCD spectra, two sets of bands are observed in the region of 7,000 and 18,000 cm^−1^. These bands can be expected to be due to the ^4^A_2_→^4^T_1_(F) and ^4^A_2_→^4^T_1_(P), respectively (*T*_d_ notation). In *D*_2d_, the two ^4^T states are split, which can be seen rather well for the lower energy transition.

However, the low-energy MCD band appears to be split into three components rather than the two expected for *D*_2d_ symmetry. Furthermore, the transition energy of the high-energy transition (18,000 cm^−1^) is larger than typically expected for tetrahedral cobalt(II) (16,000 cm^−1^)^44^. This suggests that the ligand field splitting for **1** is much larger than for typical tetrahedral cobalt(II) complexes. We have carried out a ligand field analysis in the angular overlap model (AOM) parametrization which yielded the Racah parameters *B*=653 cm^−1^, *C*=2,942 cm^−1^ and the AOM parameters *e*_σ_=6,410 cm^−1^, and *e*_πs_=1,841 cm^−1^ for sigma (*e*_σ_) and out of Co-NCCN plane π(*e*_πs_) interactions. This analysis demonstrates that symmetry lowering from *T*_d_ to *D*_2d_ strongly splits the ^4^T_2_ first excited state (*T*_d_) into ^4^E and ^4^B_2_ (*D*_2d_), where the higher ^4^E component is raised in energy to such an extent that the ^4^B_1_→^4^E(^4^T_2_) transition (*D*_2d_) moves from the mid-infrared into the near infrared and is observed in the MCD spectrum close to the two components of the electronic transition to the next higher term (Fig. 5).

We have recorded the MCD intensity at 18,100 and 18,700 cm^−1^ as a function of field at different temperatures between 1.5 and 20 K (variable-temperature-variable-field, measurement, Supplementary Fig. 7). These data can be fit rather well with a pseudo spin *S*=½ and *g*_⊥_=0, *g*_||_=9.1, using Supplementary Equation S1 (ref. 45), in good agreement with the magnetization data and correlated calculations (Fig. 2). The MCD instrument also allows us to measure a magnetic hysteresis curve by recording the MCD intensity at a given wavelength as a function of field. The inset of Fig. 5 shows that clear magnetic hysteresis is observed at 18,100 cm^−1^, *T*=1.5 K and a sweep rate of 0.5 T min^−1^, with a coercive field of *ca.* 0.24 T. To our knowledge, sizable coercivity has not been observed previously for mononuclear transition metal single-molecule magnets, with the previous highest coercive field of 5 mT at 30 mK (ref. 46). Interestingly, the coercive field is much higher than that observed in conventional SQUID measurements. One origin of this difference might lie in the slightly lower temperature (1.5 K) for the MCD measurements compared with the SQUID measurements (1.8 K). Furthermore, this difference may be due to the polarization of the electronic transition, which means that only molecules with a specific orientation with respect to the magnetic field are excited in the MCD measurement, while all molecules contribute to the SQUID-measured magnetic moment. The transition excited in the MCD-hysteresis measurement is ^4^B_1_→^4^E in *D*_2d_ symmetry notation. This transition can only be excited by the *x*,*y*-component of the electric dipole operator (which transform as *E* in *D*_2d_). The hysteresis curve displays steps, which we attribute to quantum tunnelling. However, these steps are shifted from zero field, presumably by interactions with neighbouring molecules. Such effects have also been observed in dysprosium dimers^47^.

### Theoretical calculations

To shed further light on the peculiar electronic structure, we have performed correlated calculations (CASSCF), where we have taken into account dynamic electron correlation (NEVPT2). Bond distances and angles from DFT geometry optimizations conform well to the experimental ones (Supplementary Table 2, Supplementary Fig. 8). An *ab initio* ligand field (AILFT) analysis of the CASSCF/NEVPT2 results using the crystallographically determined structure gives the following parameters: *e*_σ_=5,226 cm^−1^, *e*_πs_=1,473 cm^−1^, *B*=1,031 cm^−1^ and *C*=4,151 cm^−1^. It is apparent that the calculations underestimate the ligand field parameters by about 20%, while overestimating the Racah parameters by more than 30%. As a result, the energies for the electronic transitions to the states arising from the ^4^P free ion term are strongly overestimated (Fig. 5, Supplementary Table 3).

The calculations confirm that the bis(sulfonamide) ligand acts as both a strong σ- and a strong π-donor. This strong ligand field, together with the strong axial distortion as evidenced by the very acute N–Co–N angles (see above), leads to a rather unsual d-orbital splitting (Fig. 1), with and almost degenerate and a very small energy gap to *d*_*xy*_. The quasi-degeneracy of the former two orbitals is lifted on lowering the symmetry to *D*_2_ (Supplementary Fig. 9). Very recently, another example of a tetrahedral cobalt(II) single-ion magnet featuring an acute bite-angle ligand was reported^24^.

Projecting the lowest four states of CASSCF/NEVPT2 calculations onto an *S*=3/2 pseudo spin allows extraction of the spin-Hamiltonian parameters. The *D* value thus obtained is *D*=–112 cm^−1^, in very good agreement with the value obtained from the far-infrared and magnetization measurements. A very small *E* value of –1.1 cm^−1^ is also calculated. The very small *E* value confirms the highly axial nature of the ion, which explains the very small transition matrix elements between states with opposite projections of the magnetic moment. The two lowest KD are calculated to have the following compositions (|^2*S*+1^Γ *S m*_S_>): KD1=0.83 |^4^B_1_ 3/2 ±3/2>+0.50|^4^B_2_ 3/2 ±3/2> and KD2=0.96 |^4^B_1_ 3/2 ±1/2>−0.28 |^4^B_2_ 3/2±1/2>. Thus very strong mixing occurs between the ^4^B_1_ ground term and the very low-lying ^4^B_2_ excited term. The effective *g* values of the lowest KD projected on an *S*=1/2 pseudo spin are *g*_⊥_=0.056, *g*_||_=9.43. Calculations in the basis of the 10*S*=3/2 and 40*S*=1/2 multiplets yield susceptibility and magnetization curves that are in good agreement with the experiment (Fig. 2).

Using the DFT/BP86 optimized geometry (Supplementary Data set 2), we have also calculated the vibrational far-infrared spectrum, which shows at least three pronounced vibrational excitations in the region of the magnetic resonance transition at 230 cm^−1^ (Supplementary Fig. 6). These vibrations do not have pronounced stretching or bending behaviour of specific bonds, but all have some metal–ligand stretching character. The latter can be expected to induce modulation of the ligand field, leading to spin-phonon coupling. In this region four experimental features are observed (at 217, 222, 230 and 237 cm^−1^). Of these, the first (217 cm^−1^) is completely field independent and for the last (237 cm^−1^) only the intensity changes on application of a field. The two features at 221.7 and 229.5 cm^−1^ in zero field show a shift to higher energy reaching largest values at the highest field (*B*=11 T, 222.6 and 233.5, respectively). The presence of two rather than only one (±3/2→±1/2) field-dependent absorption points towards intermixing of spin and vibrational states which appear close in energy. We have therefore analysed the central two features in terms of a spin-vibrational coupling model. A Hamiltonian describing this coupling, considering a single effective vibrational normal mode is given in equation (2):


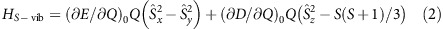


where *D* and *E* are the ZFS *D*- and *E*-terms, respectively, and *Q* is the displacement along the normal coordinate. Of the two terms in equation (2) only the former can induce magnetic resonance transitions, and the latter term was therefore not considered further in the analysis. Assigning the 222.6 and 233.5 features (*B*=11 T) to transitions dominated by the excitations of single vibrational (0→1) and magnetic (–3/2→–1/2) quanta, respectively, and employing the zero-field values for the same transitions (221.7 cm^−1^ and 229.50 cm^−1^, respectively) a fit of four model parameters to the experimental data was carried out. In this fit, an isotropic *g* value was assumed and vibronic effects on the *g* tensor were neglected. This resulted in the following set of best fit parameters: *D*=–144.5 cm^−1^, 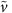
=223.1 cm^−1^, (∂*E*/∂*Q*)_0_=2.43 cm^−1^, *g*=1.24. The *g* value is clearly much lower than obtained from both the experiments as well as from the CASSCF/NEVPT2 calculations, which indicates the limitations of the very basic model that we used. The eigenfunctions obtained from the vibronic coupling analysis indeed show mixed spin/vibrational character.

The question remaining is what the origin of the very large ZFS is. The correlated calculations showed that the main contribution to the term splitting comes from the *T*_d_ and *D*_2d_ symmetry components of the ligand field and that ligand field contributions with symmetries lower than *D*_2d_ do not contribute significantly to the term splitting (Supplementary Fig. 10). In *D*_2d_ symmetry, for well-separated terms, the *D* value is related to the ligand field excitation energies as given in equation (3)^48^:


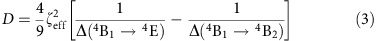


The main feature of interest for the explanation of the magnetic properties is the huge splitting (6,800 cm^−1^) of the ^4^T_2_ term (*T*_d_) by the *D*_2d_ component of the ligand field. As a result of this splitting, the ^4^B_2_ term (*D*_2d_) drops down to very low energies, in fact to only 539 cm^−1^ above the ^4^B_1_ ground term. Inserting the calculated energy gaps (539, 7,313 cm^−1^) and the effective spin-orbit coupling constant (*ζ*_eff_=446 cm^−1^) into equation (3) yields a *D* value of *D*=–152 cm^−1^, which is of the order of that found in experiment and calculation. However, the lowest energy gap (539 cm^−1^) is of the size of the spin-orbit coupling constant *ζ*_eff_. This invalidates equation (3), which is based on perturbation theory. Still, the qualitative statement that the large *D* value is due to the extremely small energy gap between ground and first excited states remains true.

## Discussion

We have presented an air- and moisture-stable mononuclear cobalt(II)-based single-molecule magnet with a very high-energy barrier towards relaxation of the magnetic moment. This energy barrier is due to the strong ligand field of the bis(sulfonamido) ligand in combination with a strong axial distortion. This leads to a very small gap between the ground and first excited states, resulting in a very large ZFS. Second, the rhombic distortion is minimal, which suppresses under-barrier relaxation processes. Complex **1** is also a highly promising building block for larger strongly exchange coupled clusters. In this respect, transition metal ions have a huge advantage over lanthanides, because strong exchange couplings are much more easily obtained. In such polynuclear clusters, quantum tunnelling is further suppressed.^11^ One could easily imagine converting the bidentate disulfonamido-benzene into a tetradentate bridging ligand. Such ligands are also redox active, where very strong exchange couplings are expected for the bridging ligand in a radical form.

## Methods

### Synthesis and sample preparation

Complex **1** was synthesized as follows: H_2_L (212 mg, 0.80 mmol, 2 equiv.) and Co(BF_4_)_2_ 6 H_2_O (136 mg, 0.40 mmol, 1 eqequiv.) were dissolved in acetonitrile (10 ml) and NEt_3_ (0.5 ml) was added. The reaction mixture was stirred at room temperature. Diffusion of diethylether into the acetonitrile solution yielded pink crystals (250 mg, 0.32 mmol, 80%) of the desired complex. Those were also suitable for X-ray analysis. Elem. Anal. Calc. for C_28_H_50_CoN_6_O_8_S_4_ 0.15 H_2_O C 42.65; H 6.41; N 10.66% found C 42.66; H 6.75; N 10.63%. ESI-MS calc. for C_16_H_23_CoN_4_O_8_S_4_ (M – 2 HNEt_3_+3 H^+^): *m/z* 585.9731 found 585.9713.

Elemental analysis was performed on a Perkin Elmer Analyser 240. Mass spectrometry experiments were carried out on a Bruker Daltronics Mictrotof-Q mass spectrometer.

Single crystals of **1** were grown by the slow diffusion of diethylether into an acetonitrile solution. The X-ray diffraction measurement was performed on a BRUKER Smart AXS diffractometer (graphite-monochromated Mo Kα radiation, *λ*=0.71073 Å). SHELXS-97 and SHELXL-97 were used to solve and refine the structure^49^. The CCDC deposition number is CCDC 971167.

### Physical measurements

Magnetic measurements were carried out on teflon-wrapped pressed powder pellets in applied fields of 1,000–10,000 Oe with a Quantum Design MPMS3 SQUID magnetometer. The data were corrected for the diamagnetic contribution to the magnetic susceptibility by means of Pascal's constants. Far-infrared spectra were recorded on pressed powder pellets of **1** dispersed in eicosane on a Bruker IFS 66v/s FTIR spectrometer with a globar source, where the sample was placed inside an 11 T solenoid magnet, with a composite bolometer detector element located inside the magnet. MCD measurements were recorded on a spectrometer built around an Aviv 42 CD spectrometer equipped with both photomultiplier and InGaAs detectors and an Oxford Instruments Spectromag SM4000 optical cryomagnet, allowing measurements at wavelengths between 250 and 2,000 nm, at temperatures down to 1.5 K and fields up to 10 T. High-frequency EPR (HFEPR) spectra were recorded on a home-built spectrometer featuring an Anritsu signal generator, a VDI amplifier-multiplier chain, a Thomas Keating quasi-optical bridge, an Oxford Instruments 15/17 T solenoid cryomagnet and a QMC Instruments InSb hot electron bolometer.

### Data analysis and simulation.

For susceptibility and FIR simulations, the energy levels were calculated by means of the Easyspin toolbox for Matlab^50^. These were then used to calculate the susceptibility numerically.

Ligand field parameters have been fitted directly to the experimental *d–d* transition energies by means of the AOMX programme^51^. In these fits, we have used the crystal structure. The fit was performed in three steps; in the first step, the parameters *e*_σ_ and *e*_πs_ and *B* were obtained from the positions of the spin-allowed *d–d* transitions at 6,211, 7,236, 8,217, 17,952 and 18,911 cm^−1^. In a second step, adopting the resulting values of the parameters *e*_σ_ and *e*_πs_ and *B* we obtained the value of *C* from a best fit to the energies of the two experimental spin-forbidden transitions (16,156 and 16,811 cm^−1^). Finally, the spin-orbit coupling parameter was fitted from the energy of the transition (–2*D*) leaving all other parameters (*e*_σ_, *e*_πs_, *B* and *C*) unchanged.

### Calculations

Correlated calculations were carried out on the complex geometry from X-ray data and on BP86 DFT-optimized structure, by using the ORCA programme suite^52^,^53^. (Supplementary Table 2). Complete active space self-consistent field (CASSCF) and the N-electron-valence-perturbation theory to second order (NEVPT2) including the 10*S*=3/2 and 40*S*=1/2 were carried out according to the computational protocol used recently^54^. Results from the multiplet calculations are included in Supplementary Table 3. The ligand field analysis of the *ab initio* data consisted of two steps. In the first step, we derived the 5 × 5 ligand field matrix equation (4) in the basis of the five MOs of 3d type and Racah parameters of interelectronic repulsion *B* and *C*. This is a non-relativistic (spin-free) calculation.


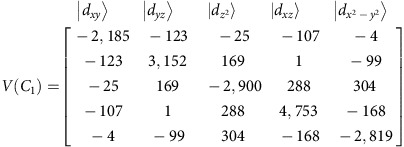


Spin-orbit coupling was accounted for within the basis (‘state interaction') of the non-relativistic CI Eigen states using quasi-degenerate perturbation theory. It allows to additionally obtain the spin-orbit sublevels and the Zeeman Hamiltonian within the same non-relativistic CI basis. From these calculations and after subtracting the diagonal non-relativistic matrix, the effective spin-orbit coupling parameter *ζ* was obtained from a best fit to both the high (*S*=3/2) and low (*S*=1/2) spin multiplets. In step 2, the 5 × 5 one-electron ligand field matrix was fitted with the parameters *e*_σ_ and *e*_π*s*_, quantifying Co-N σ and π-antibonding interactions, respectively. They are defined following the AOM, which accounts for the coordination geometry and thus makes these parameters characteristic for a particular metal–ligand bond. Ligand field parameters derived for **1**, based on both the X-ray and the BP86 DFT-optimized geometries are collected in Supplementary Table 4. Diagonalization of the 5 × 5 ligand field matrix yields the ligand field orbital splitting diagram (Supplementary Fig. 9) and a factoring of the ligand field into increments due to a successive symmetry lowering from spherical (*R*_3_) to *T*_d_ to *D*_2d_ and *C*_1_ symmetries yields term splitting diagrams of the Co^2+^ ion in the complex (Supplementary Fig. 10). Finally, parameters of the effective spin-Hamiltonian and main values of the *g* tensor of the ground KD are listed in Supplementary Table 5.

For the analysis of ligand field splitting of the d-orbitals, the AILFT (*ab initio* ligand field theory) programme was used.^55^,^56^

To analyse the phenomenon of spin-vibronic coupling, we have computed the full vibrational spectrum of the complex. In this calculation, we have used the DFT-BP86-optimized geometry. In the range of the magnetic peak at 230 cm^−1^ as many as eight vibrational modes are computed (Supplementary Fig. 6a). The spin-Hamiltonian of the *S*=3/2 system can be expanded into a Taylor series around the reference geometry with a zero-order axially symmetric term equation (5) and a term that is linearly dependent on the normal modes equation (6)


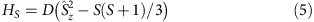



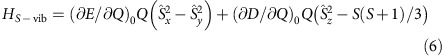


Here we restrict the consideration to a single effective interacting mode *Q*.

With 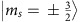 and 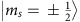 as functions for the S=3/2 spin and 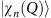
(n-the vibrational quantum number) the Harmonic oscillator wavefunctions, the spin-vibronic wavefunction can be expanded into a series of products of uncoupled spin and vibrational functions:


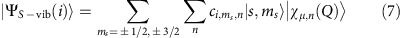


The total Hamiltonian *H*_eff_ equation 10 consists of the terms of equations (5) and (6) and, in addition of the harmonic oscillator equation (8), *ħω* is the vibrational energy quantum and the Zeeman equation (9) Hamiltonians:


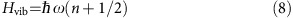



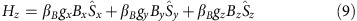



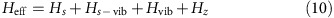


The ground state of the system without a magnetic field is 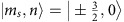 while the lowest excited states are 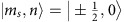 and 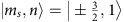. They correspond to a magnetic excitation and to the excitation of one vibrational quantum, respectively. Since −2*D*≈*ħω*≈230 cm^−1^, the two excited states are close in energy and interact with each other via the matrix element equation (11).


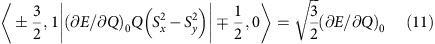


When switching on the magnetic field the spin-states split and terms of the type of equation (11) lead to their mixing. Vibrational excitation of the type 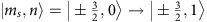 should not depend on the magnetic field but because of their mixing with magnetic excitations that do depend on the field (via terms of the type of equation (11)) they start sharing field dependence with them, that is with the 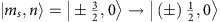 field-dependent transitions. Restricting to a weak vibronic coupling and taking only the ground and first excited state Harmonic oscillator eigenfunctions *n*=0,1, we list in Supplementary Table 6 the spin-vibronic Hamiltonian of the *S*=3/2 spin system coupling to one vibrational mode only. We further, based on the discussion in the text, identified the 221.7 and 229.5 cm^−1^ transitions (*B*=0) and the ones 222.6 and 233.5 cm^−1^ (*B*=11 T) with transitions from the ground spin-vibronic state into the 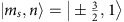, 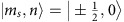, 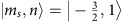 and 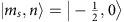 excited states, respectively. We note that without spin-vibronic mixing and without a magnetic field only transitions from the 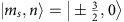 ground state into the 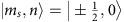 and 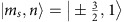 states are formally allowed. These correspond to the 221.7 and 229.5 cm^−1^ features in the experimental spectrum. As seen from Supplementary Table 7, owing to the proximity of the two states, spin-vibronic coupling considerably mixes the one state into the other (18%). A field of *B*=11 T substantially modifies the wavefunctions of these states; it increases the *m*_*s*_=–3/2 spin character of the former state (nominally an excitation of one vibrational quantum) and the *m*_*s*_=–1/2 character of the latter state (nominally a pure *m*_*s*_=–3/2 to *m*_*s*_=–1/2 spin excitation). It is impressive that all other states computed at 241,40, 243.39 being formally forbidden, both by spin and by vibrational quantum numbers become partially allowed due to modification of their functions by the magnetic field. However, these modifications are small (see Supplementary Table 7). Thus, within the confines of the approximations inherent in the basic model, one can also explain why out of the six possible spin-vibronic transitions only two show up with considerable intensity in the spectrum.

## 

## Additional information

**Accession codes:** The X-ray crystallographic coordinates for structures reported in this study have been deposited at the Cambridge Crystallographic Data Centre (CCDC), under deposition number CCDC 971167. These data can be obtained free of charge from The Cambridge Crystallographic Data Centre via www.ccdc.cam.ac.uk/data_request/cif.

**How to cite this article:** Rechkemmer, Y. *et al*. A four-coordinate cobalt(II) single-ion magnet with coercivity and a very high energy barrier. *Nat. Commun.* 7:10467 doi: 10.1038/ncomms10467 (2016).

## Supplementary Material

Supplementary Information Supplementary Figures 1-10, Supplementary Tables 1-7 and Supplementary Reference

Supplementary Data 1 cif file of the title compound

Supplementary Data 2 Input for DFT calculation and calculated vibrational spectrum.

## Figures and Tables

**Table 1:** Selected examples for zero-field splitting parameters.

**Compound**	***D*** **(cm**^–1^**)**	***U***_**eff**_ **(cm**^–1^**)**	**Literature**
(Ph_4_P)_2_[Co(C_3_S_5_)_2_]	–161	33.9	Fataftah *et al*.^19^
(HNEt_3_)_2_[Co(pdms)_2_]	–115	118	*
(Ph_4_P)_2_[Co(SePh)_4_]	–83	19.1	Zadrozny *et al*.^20^
[Co(AsPh_3_)_2_(I)_2_]	–74.7	32.6	Saber *et al*.^21^
[Co(salbim)_2_]	+67	—	Šebová *et al*.^22^
(Ph_4_P)_2_[Co(SPh)_4_]	–62	21.1	Zadrozny *et al*.^20^
[Co{NtBu)_3_SMe}_2_]	–58	75†	Carl *et al*.^24^
[Co(acac)_2_(H_2_O)_2_]	+57	—	Gómez-Coca *et al*.^23^

Reported zero-field splitting *D*-values with |*D*|>50 cm^−1^ and relaxation energy barriers *U*_eff_ of tetrahedral cobalt(II) complexes.

^*^This work

^†^In a 1,500 Oe applied magnetic field.
