# Supplementary material for: A four-coordinate cobalt(II) single-ion magnet with coercivity and a very high energy barrier
Source: Nat Commun. 2016 Feb 17;7:10467. doi: 10.1038/ncomms10467 (PMC4757785; doi:10.1038/ncomms10467)
Supplement: Supplementary Information — Supplementary Figures 1-10, Supplementary Tables 1-7 and Supplementary Reference [file ncomms10467-s1.pdf]

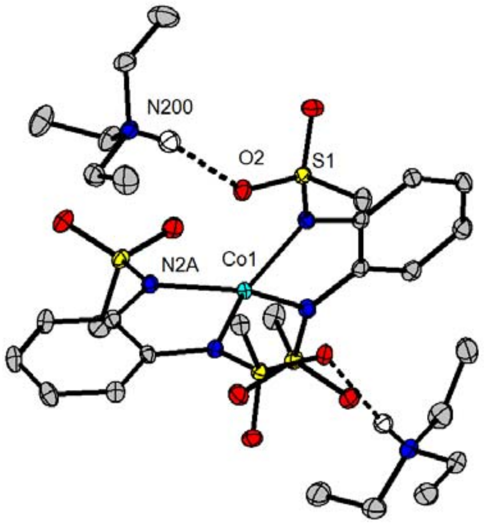

Supplementary Figure 1 | ORTEP plot of 1. Co is shown in cyan, N blue, S yellow, O red and C grey. Ellipsoids drawn at 50% electron density. Hydrogen atoms and solvent molecules have been omitted for clarity except for the hydrogen of the counter ion $HNEt_3^+$, which exhibits a hydrogen bond with the ligand that is denoted as dashed line.

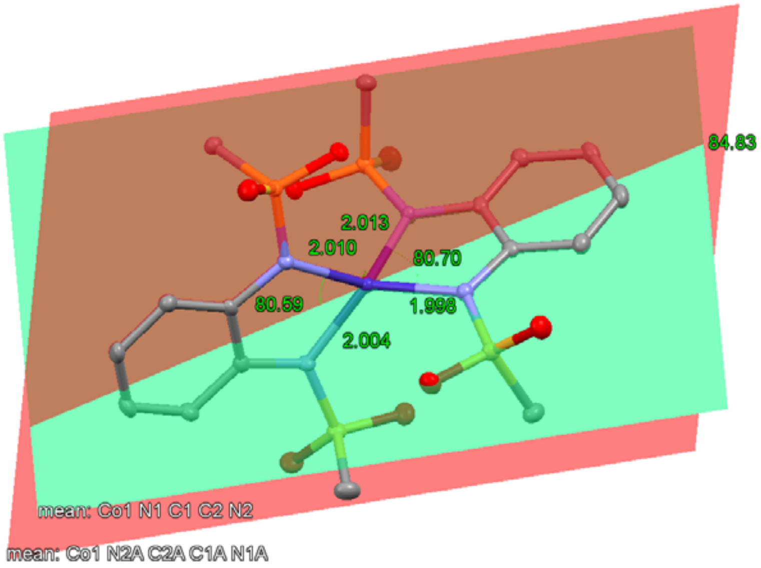

Supplementary Figure 2 | Crystallographically determined structure of 1. Displayed are the two planes defined by the two Co-NCCN metallacycles, the angle between them, as well as selected bond distances and angles.

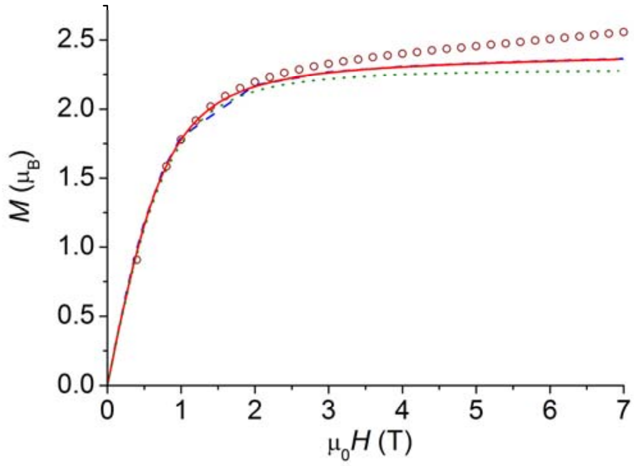

Supplementary Figure 3 | Magnetisation. Magnetisation as a function of applied field at T = 1.8 K recorded on a powder sample of **1**. The solid red line is a spin Hamiltonian fit with D = –115 cm^{-1}, $g_\perp$ = 2.20, $g_{||}$ = 3.03, the dashed blue line is a simulation on the basis of the correlated calculations and the dotted green line is a fit of the magnetisation using pseudo spin S = ½ and $g_\perp$ = 0, $g_{||}$ = 9.1.

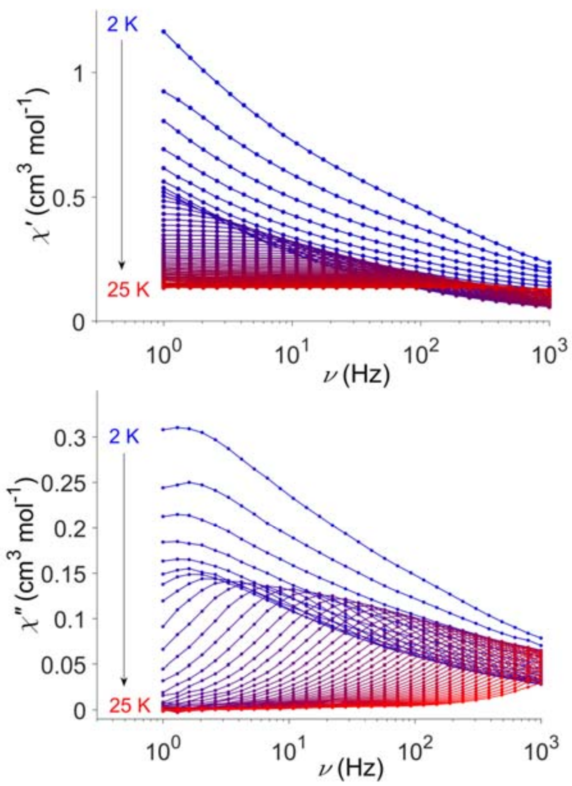

Supplementary Figure 4 | AC susceptibility vs. frequency. In-phase χ' and out-of-phase χ'' components of the susceptibility as a function of frequency recorded on a pressed powder sample of **1** in zero dc field and at different temperatures as indicated.

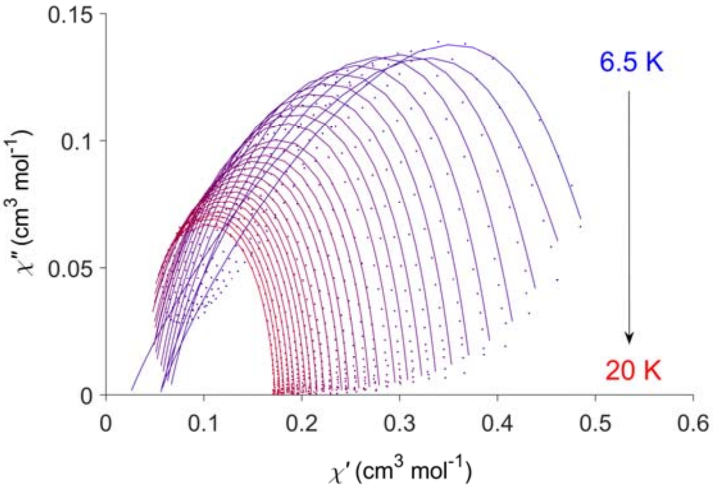

Supplementary Figure 5 | Argands plots. Argand plots derived from the frequency-dependent ac susceptibility data at various temperatures, as indicated. Solid lines correspond to best fits.

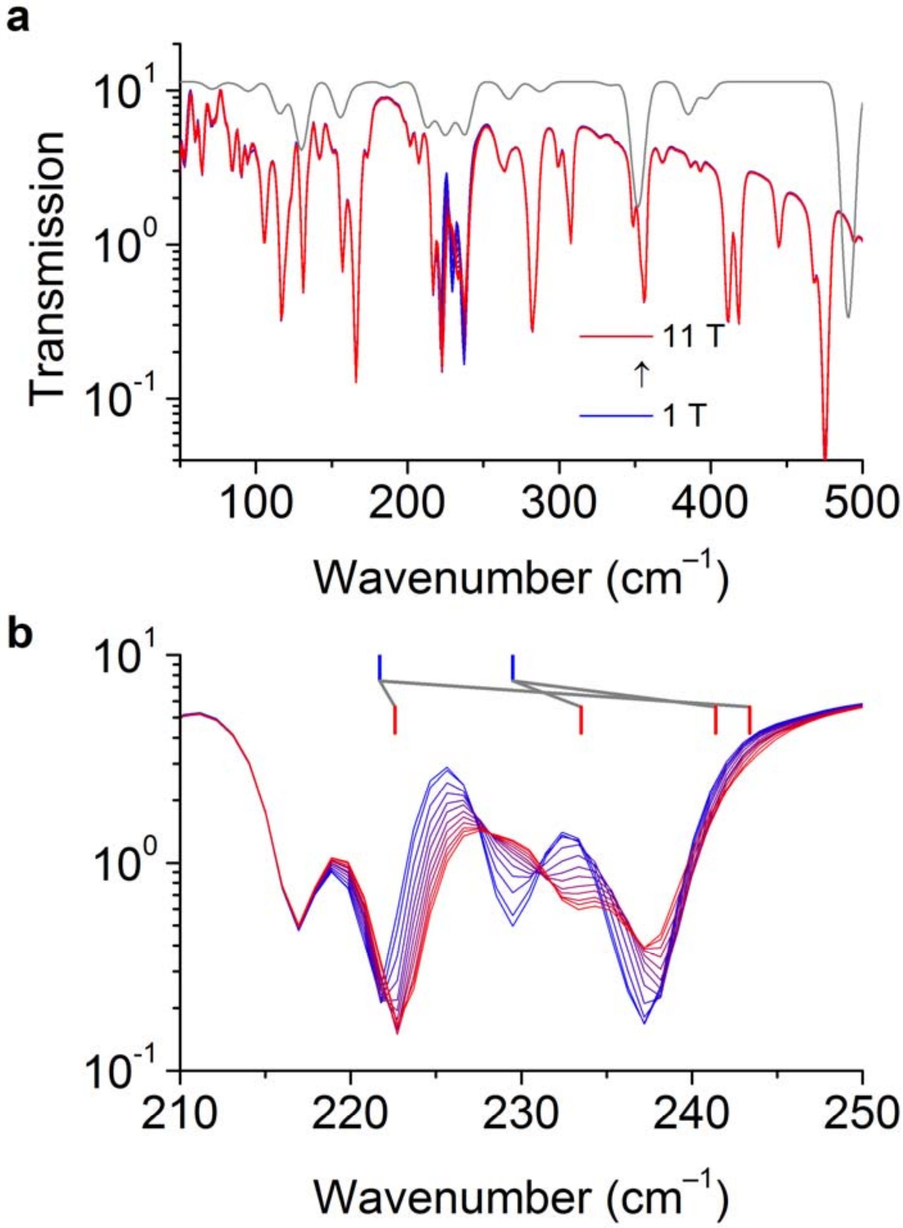

Supplementary Figure 6 | Far infrared measurements. (a) Far infrared transmission spectra recorded on a pressed powder pellet of **1** at T = 4 K and different fields as indicated. The grey line is the vibrational spectrum calculated by DFT using the BP86 optimized geometry. (b) Enlargement of the field-dependent features. The blue and red lines indicate the calculated 0 T and 11 T excitation energies resulting from the spin-vibrational analysis.

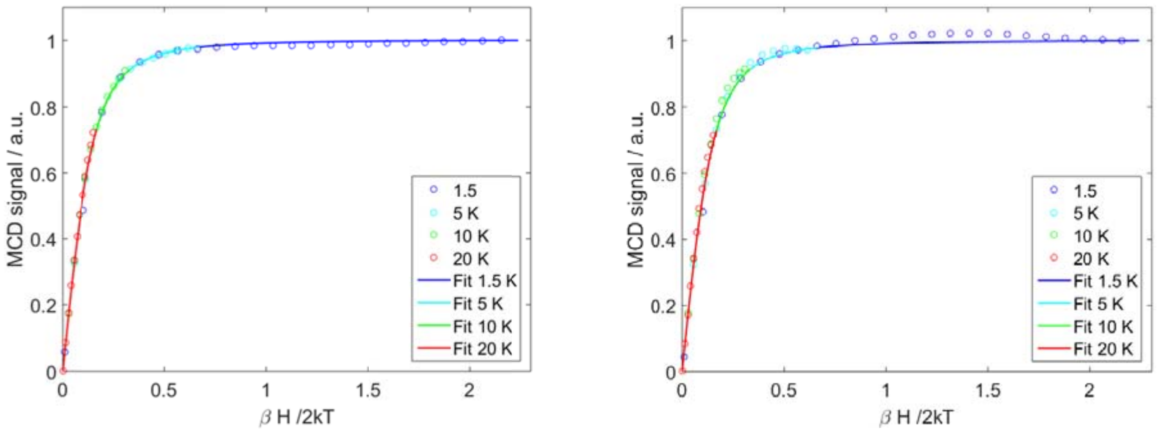

Supplementary Figure 7 | Magnetic Circular Dichroism. VTVH data recorded on a mull of 1 at 553 nm (18083 cm^{-1}) and 536 nm (18657 cm^{-1}).

The VTVH data were normalised by the saturation magnetisation and fitted with the following formula:[1]

$$\frac{\Delta\varepsilon_{\mathrm{av}}}{E} = -\frac{\gamma}{4\pi}\int_{\theta}\int_{\varphi}\tanh\left(\frac{g\mu_{\mathrm{B}}B}{2kT}\right)\frac{\sin\theta}{g}\left(l_x^2 g_x M_{yz}^{eff} + l_y^2 g_y M_{xz}^{eff} + l_z^2 g_z M_{xy}^{eff}\right)\mathrm{d}\varphi\mathrm{d}\theta \qquad \text{eq. S1}$$

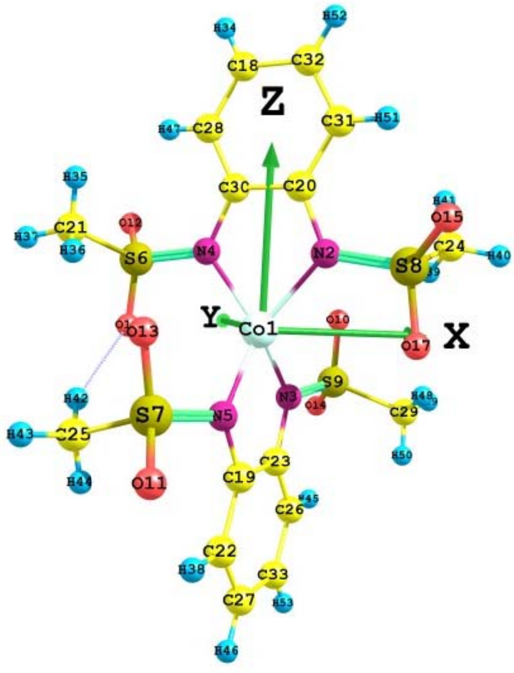

Supplementary Figure 8 | Numbering for calculations. Complex **1** with atom numbering and the choice of Cartesian axes.

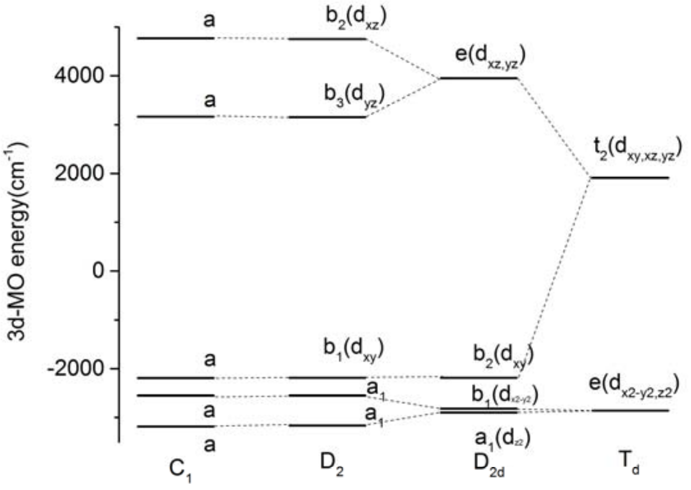

Supplementary Figure 9 | MO diagram. Ab-initio 3d-MO ligand field diagrams from a mapping of ligand field theory on CASSCF/NEVPT2 eigenvectors/eigenvalues for a successive lowering of symmetry from T_d to D_{2d} to D_2 to C_1.

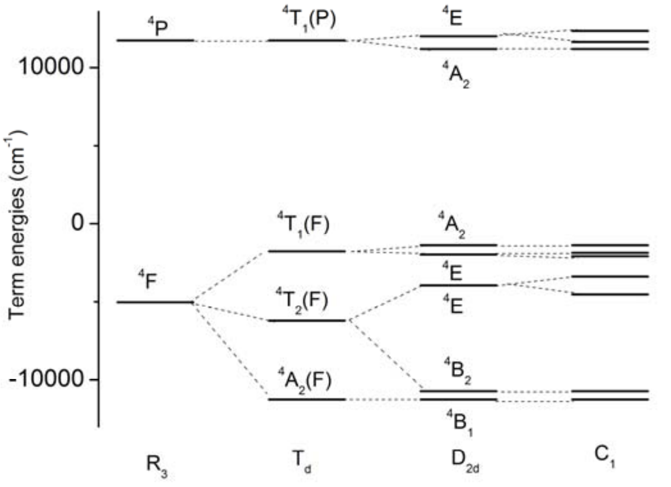

Supplementary Figure 10 | Term diagram. Term energy diagram and contributions of the ligand field to the term splitting due to the successive symmetry lowering from spherical R_3 to T_d to D_{2d} and C_1 point groups.

Supplementary Table 1 | Details of the crystallographic structure determination of 1.

	**1**
Chemical formula	$C_{28} H_{52} Co N_6 O_8 S_4$
M_r	787.93
Crystal system	orthorhombic
Space group	$P2_12_12_1$
a (Å)	12.153(2)
b (Å)	15.986(3)
c (Å)	18.290(3)
α (°)	90
β (°)	90
γ (°)	90
V (Å^3)	3553.3(11)
Z	4
D_{calc} (g/cm^3)	1.473
Temperature (K)	100(2)
μ (mm^{-1})	0.773
Crystal size (mm)	0.60 x 0.55 x 0.30
*K*α	0.71073 (Mo)
F(000)	1668
meas./ indep. refl.	47602/8175
obsvd. [I > 2σ(I)] refl.	7978
R_{int}	0.0176
$R[F^2 > 2\sigma(F^2)]$	0.0201
w*R* (F^2)	0.0527
S	1.032
$\Delta\rho_{max}$, $\Delta\rho_{min}$ (e Å^{-3})	0.304, -0.268

Supplementary Table 2 Co-N bond distances and N-Co-N bond angles **1** from X-ray data and from the BP86/DFT geometry optimization.[a]

Bond distance(Å)/angle (o)	X-ray geometry	DFT/BP86 geometry
Co1N2	2.012	1.970
Co1N4	1.998	1.968
Co1N3	2.010	1.967
Co1N5	2.004	1.968
∠N2Co1N4	80.7	83.0
∠N2Co1N4	80.6	82.9
∠N2Co1N4	121.4	123.7
∠N2Co1N4	128.0	124.4
∠N2Co1N4	128.0	124.7
∠N2Co1N4	124.8	123.8
θ	80.65	82.95
2ω	85.2	89.2

[a] see Supplementary Figure 7 for numbering of atoms.

Supplementary Table 3 Energies (in cm^{-1}) of spin-free S = 3/2 states spanned by the d^7 configuration in **1**, their assignment to the constituting strong field electronic configurations and oscillator strengths of electronic transitions from the $^4B_1[^4A_2(^4F)]$ ground state to the S = 3/2 excited states from NEVPT2 calculations using the X-ray-determined structure geometry. [a]

Electronic state $D_{2d}[T_d(R_3)]$	Energy(cm^{-1})	Strong Field Electronic Configurations	f.10^5
$^4B_1[^4A_2(^4F)]$	0 (0)	1.00 $d_{xy}^1 d_{yz}^1 d_{z2}^2 d_{xz}^1 d_{x2-y2}^2$	-
$^4B_2[^4T_2(^4F)]$	539(689)	1.00 $d_{xy}^2 d_{yz}^1 d_{z2}^2 d_{xz}^1 d_{x2-y2}^1$	1.35
$^4E[^4T_2(^4F)]$	6744(7981)	0.75 $d_{xy}^1 d_{yz}^2 d_{z2}^1 d_{xz}^1 d_{x2-y2}^2$;0.25 $d_{xy}^1 d_{yz}^2 d_{z2}^2 d_{xz}^1 d_{x2-y2}^1$	2.55
	7881(9338)	0.80 $d_{xy}^2 d_{yz}^2 d_{z2}^1 d_{xz}^1 d_{x2-y2}^1$;0.10 $d_{xy}^1 d_{yz}^1 d_{z2}^2 d_{xz}^2 d_{x2-y2}^1$ 0.10 $d_{xy}^1 d_{yz}^1 d_{z2}^1 d_{xz}^2 d_{x2-y2}^2$	3.49
$^4E[^4T_1(^4F)]$	9193(10148)	0.76 $d_{xy}^2 d_{yz}^1 d_{z2}^1 d_{xz}^2 d_{x2-y2}^1$;0.18 $d_{xy}^1 d_{yz}^2 d_{z2}^2 d_{xz}^1 d_{x2-y2}^1$	1.54
	9402(10200)	0.71 $d_{xy}^1 d_{yz}^1 d_{z2}^1 d_{xz}^2 d_{x2-y2}^2$;0.28 $d_{xy}^1 d_{yz}^1 d_{z2}^2 d_{xz}^2 d_{x2-y2}^1$	0.80
$^4A_2[^4T_1(^4F)]$	9890(11043)	0.54 $d_{xy}^2 d_{yz}^1 d_{z2}^1 d_{xz}^1 d_{x2-y2}^2$;0.44 $d_{xy}^1 d_{yz}^2 d_{z2}^1 d_{xz}^2 d_{x2-y2}^1$	1.83
$^4A_2[^4T_1(^4P)]$	22466(23807)	0.55 $d_{xy}^1 d_{yz}^2 d_{z2}^1 d_{xz}^2 d_{x2-y2}^1$;0.45 $d_{xy}^2 d_{yz}^1 d_{z2}^1 d_{xz}^1 d_{x2-y2}^2$	
$^4E[^4T_1(^4P)]$	22920(23643)	0.57 $d_{xy}^1 d_{yz}^2 d_{z2}^2 d_{xz}^1 d_{x2-y2}^1$;0.23 $d_{xy}^2 d_{yz}^1 d_{z2}^1 d_{xz}^2 d_{x2-y2}^1$; 0.19 $d_{xy}^1 d_{yz}^2 d_{z2}^1 d_{xz}^1 d_{x2-y2}^2$	38.78
	23622(23695)	0.62 $d_{xy}^1 d_{yz}^1 d_{z2}^2 d_{xz}^2 d_{x2-y2}^1$;0.20 $d_{xy}^1 d_{yz}^1 d_{z2}^1 d_{xz}^2 d_{x2-y2}^2$; 0.18 $d_{xy}^2 d_{yz}^2 d_{z2}^1 d_{xz}^1 d_{x2-y2}^1$	39.32

[a] Energies computed when using NEVPT2 along with the DFT optimized geometry are listed in parentheses.

Supplementary Table 4 Ab initio (CASSCF/NEVPT2) ligand field parameters (in cm^{-1}) for **1** in its X-ray and BP86 DFT optimized geometries. The parameters e_σ and $e_{\pi s}$ derive from the angular overlap model formalism and parametrize σ and π metal-ligand interactions, respectively; B and C are Racah parameters; ζ is the effective spin-orbit coupling constant.

Geometry	e_σ	$e_{\pi s}$	B	C	ζ
X-ray	5226	1473	1031	4151	446
BP86 DFT	5578	1623	1020	4108	446

Supplementary Table 5 Parameters of the Spin-Hamiltonian *D*, *E*, g_i (i = x,y,z) and pseudo-spin ½ *g*-tensor components of the ground Kramers doublet (KD) of **1** from QDPT spin-orbit coupling calculations utilizing the CASSCF CI eigenvectors as a non-relativistic basis and NEVPT2 corrected energy eigenvalues.

Parameters of the effective Spin Hamiltonian					Ground Kramers Doublet g-tensor components		
D	E/D	g_x	g_y	g_z	g_x^{KD}	g_y^{KD}	g_z^{KD}
-111.9	0.01	2.005	1.959	3.234	0.056	0.055	9.430

Supplementary Table 6 The spin-vibronic Hamiltonian in the basis of the products of the S=3/2 spin (±3/2,±1/2) and ground/first excited state vibrational functions (n=0/1).

	$\left\|\frac{3}{2},0\right\rangle$	$\left\|\frac{1}{2},0\right\rangle$	$\left\|-\frac{1}{2},0\right\rangle$	$\left\|-\frac{3}{2},0\right\rangle$	$\left\|\frac{3}{2},1\right\rangle$	$\left\|\frac{1}{2},1\right\rangle$	$\left\|-\frac{1}{2},1\right\rangle$	$\left\|-\frac{3}{2},1\right\rangle$
$\left\|\frac{3}{2},0\right\rangle$	$D+\frac{\hbar\omega}{2}+\frac{3}{2}G_{zz}$	$\frac{\sqrt{3}}{2}(G_{xx}-iG_{yy})$	0	0	$\frac{1}{\sqrt{2}}D'$	0	$\sqrt{\frac{3}{2}}E'$	0
$\left\|\frac{1}{2},0\right\rangle$	$\frac{\sqrt{3}}{2}(G_{xx}+iG_{yy})$	$-D+\frac{\hbar\omega}{2}+\frac{1}{2}G_{zz}$	$(G_{xx}-iG_{yy})$	0	0	$-\frac{1}{\sqrt{2}}D'$	0	$\sqrt{\frac{3}{2}}E'$
$\left\|-\frac{1}{2},0\right\rangle$	0	$(G_{xx}+iG_{yy})$	$-D+\frac{\hbar\omega}{2}-\frac{1}{2}G_{zz}$	$-\frac{\sqrt{3}}{2}(G_{xx}-iG_{yy})$	$\sqrt{\frac{3}{2}}E'$	0	$-\frac{1}{\sqrt{2}}D'$	0
$\left\|-\frac{3}{2},0\right\rangle$	0	0	$-\frac{\sqrt{3}}{2}(G_{xx}+iG_{yy})$	$D+\frac{\hbar\omega}{2}-\frac{3}{2}G_{zz}$	0	$\sqrt{\frac{3}{2}}E'$	0	$\frac{1}{\sqrt{2}}D'$
$\left\|\frac{3}{2},1\right\rangle$	$\frac{1}{\sqrt{2}}D'$	0	$\sqrt{\frac{3}{2}}E'$	0	$D+\frac{3\hbar\omega}{2}+\frac{3}{2}G_{zz}$	$\frac{\sqrt{3}}{2}(G_{xx}-iG_{yy})$	0	0
$\left\|\frac{1}{2},1\right\rangle$	0	$-\frac{1}{\sqrt{2}}D'$	0	$\sqrt{\frac{3}{2}}E'$	$\frac{\sqrt{3}}{2}(G_{xx}+iG_{yy})$	$-D+\frac{3\hbar\omega}{2}+\frac{1}{2}G_{zz}$	$(G_{xx}-iG_{yy})$	0
$\left\|-\frac{1}{2},1\right\rangle$	$\sqrt{\frac{3}{2}}E'$	0	$-\frac{1}{\sqrt{2}}D'$	0	0	$(G_{xx}+iG_{yy})$	$-D+\frac{3\hbar\omega}{2}-\frac{1}{2}G_{zz}$	$\frac{\sqrt{3}}{2}(G_{xx}-iG_{yy})$
$\left\|-\frac{3}{2},1\right\rangle$	0	$\sqrt{\frac{3}{2}}E'$	0	$\frac{1}{\sqrt{2}}D'$	0	0	$\frac{\sqrt{3}}{2}(G_{xx}+iG_{yy})$	$D+\frac{3\hbar\omega}{2}-\frac{3}{2}G_{zz}$

Notations: $D'=(\partial D/\partial Q)_o$; $E'=(\partial E/\partial Q)_o$; $G_{xx}=\beta_B g_x B_x$; $G_{yy}=\beta_B g_y B_y$; $G_{zz}=\beta_B g_z B_z$;

$\beta_B = 0.4669$ cm^{-1}/T – the Bohr magneton.

Supplementary Table 7 Eigenvalues and composition (dominant contributions only are given) of spin-vibronic energy eigenvalues for the complex.[a]

B=0 T		B=11 T		
Energy (cm^{-1})	Wavefunction composition	Energy (cm^{-1})	Wavefunction composition	
0	100% $\left\|\frac{3}{2},0\right\rangle$	0	100% $\left\|-\frac{3}{2},0\right\rangle$	
0	100% $-\left\|\frac{3}{2},0\right\rangle$	19.20	100% $\left\|\frac{3}{2},0\right\rangle$	
221.70	82% $\left\|\frac{3}{2},1\right\rangle$,18% $\left\|-\frac{1}{2},0\right\rangle$	222.60	97% $\left\|-\frac{3}{2},1\right\rangle$, 3% $\left\|\frac{1}{2},0\right\rangle$	97% vibr. allowed
221.70	82% $\left\|-\frac{3}{2},1\right\rangle$,18% $\left\|\frac{1}{2},0\right\rangle$	233.50	90% $\left\|-\frac{1}{2},0\right\rangle$,10% $\left\|\frac{3}{2},1\right\rangle$	90% spin allowed
229.50	82% $\left\|-\frac{1}{2},0\right\rangle$,18% $\left\|\frac{3}{2},1\right\rangle$	241.40	97% $\left\|\frac{1}{2},0\right\rangle$, 3% $\left\|-\frac{3}{2},1\right\rangle$	3% vibr.allowed
229.50	82% $\left\|\frac{1}{2},0\right\rangle$,18% $\left\|-\frac{3}{2},1\right\rangle$	243.39	90% $\left\|\frac{3}{2},1\right\rangle$, 10% $\left\|-\frac{1}{2},0\right\rangle$	10% spin allowed
451.20	100% $\left\|-\frac{1}{2},1\right\rangle$	457.60	100% $\left\|-\frac{1}{2},1\right\rangle$	forbidden
451.20	100% $\left\|\frac{1}{2},1\right\rangle$	464.00	100% $\left\|\frac{1}{2},1\right\rangle$	forbidden

[a] Computed using parameters from a best fit to the experimental transition (221.7, 229.50 cm^{-1}, *B*=0 T and 222.6, 233.5 cm^{-1}, *B*=11T).

Supplementary Reference

1. Neese F, Solomon EI. MCD C-term signs, saturation behavior, and determination of band polarizations in randomly oriented systems with spin S >= 1/2. Applications to S = 1/2 and S = 5/2. *Inorg. Chem.* 1999, **38**(8)**:** 1847-1865.
